# National Clinician Survey of Outcomes for Trials in Surgical Wounds Healing by Secondary Intention (SWHSI)

**DOI:** 10.1111/iwj.71066

**Published:** 2026-10-20

**Authors:** Misha Sidapra, Judith Long, Louise Hitchman, Jane Nixon, Paul Tiffin, Catherine Arundel, Ian C. Chetter

**Affiliations:** ^1^ Academic Vascular Surgery Unit Hull York Medical School, University of Hull Hull UK; ^2^ Hull University Teaching Hospitals NHS Trust Hull UK; ^3^ Leeds Institute of Health Sciences University of Leeds Leeds UK; ^4^ Hull York Medical School University of York York UK; ^5^ York Trials Unit, Department of Health Sciences University of York York UK

**Keywords:** outcome assessment, secondary intention, surgical wound, surveys and questionnaires

## Abstract

Surgical wounds healing by secondary intention are often complex, costly and slow to heal. Consistent outcome reporting ensures that interventional trials are directly comparable and less vulnerable to reporting bias, thereby enhancing the efficiency of evidence generation. Currently, no framework for outcome reporting from interventional studies of SWHSI exists. We present a national survey of clinicians as the first step to producing a core outcome set for studies involving interventions for this wound type. A pre‐piloted online survey was administered in September 2024 via the Qualtrics system, comprising three sections: perceptions, decision‐making and key outcomes in SWHSI management. Recruitment was via professional organisations, membership mailing lists and social media. One hundred and two clinician responses were received. The most common reasons reported for choosing secondary intention healing were concerns about infection (46%, 35/76), primary wound closure (48.7%, 37/76) and potential reintervention (89.5%, 68/76). All‐cause mortality at 30 days (73%, 49/67) and 1 year (70%, 47/67), wound healing at 30 days (100%, 67/67), surgical site infection (98%, 66/67), pain score (94%, 63/67), wound size (94%, 63/67), quality of life (99%, 66/67), length of hospital stay (85%, 57/67) and reintervention requirement (90%, 60/67) were reported to be important clinical trial outcomes in this population. This survey explored the main decision‐driving factors in choosing secondary intention healing and identified important trial outcomes for clinicians in this research area.

## Key Points

- Standardisation of outcome reporting can improve the quality and utility of clinical research when developed in collaboration with key stakeholders, including clinicians, patients and researchers.
- Respondents in this UK‐based clinician survey identified infection risk, viability of primary closure and risk of re‐intervention as key concerns in their perceptions of and decision‐making around SWHSI.
- Mortality, wound healing, surgical site infection, wound size, pain, quality of life, length of hospital stay and reintervention requirement were identified as important clinical trial outcomes by respondents.
- Together with further engagement with other key stakeholder groups, this work will inform the development of a COS to standardise outcome reporting in SWHSI research.

## 1 Introduction

Surgical wounds healing by secondary intention (SWHSI) are a serious issue for patients and healthcare providers internationally [1]. Following a surgical operation, incisions are usually closed with surgical stitches or staples, known as healing by primary intention. However, in some instances a wound which has been primarily closed re‐opens or it is not possible or advisable to close a wound, and the open wound is allowed to heal from the base up. This is known as healing by secondary intention [2]. However, SWHSI can be challenging and resource‐intensive to manage with a substantial negative impact on the quality of life of affected patients [3, 4].

The annual cost of treating wounds to the NHS is estimated at £5.6bn, with complex and slow‐healing wounds like SWHSI making up a large proportion of this expenditure [5]. It is estimated that SWHSIs constitute up to 28% of all acute wounds receiving wound care [6]. There is thus an imperative to improve the clinical and cost‐effectiveness of wound treatments. In order to efficiently develop an evidence base for wound healing adjuncts there needs to be collaboration across institutions, populations and countries, and heterogeneity in outcome reporting leads to difficulty in the evidence synthesis process and a greater vulnerability to reporting bias [7]. Core Outcome Sets (COS) can be helpful in this regard. COS are co‐created groups of minimum outcomes that can be integrated into research designs to standardise outcome reporting in a research area, and allow the uncertainty associated with different treatment approaches to be more rapidly reduced as part of the evidence generation and synthesis process [7].

To develop a COS for SWHSI, input from clinicians is required particularly in relation to perceptions of SWHSI, decisions surrounding SWHSI and which trial outcomes are perceived as most important. This study therefore aimed to collate this information to inform subsequent COS development.

## 2 Methods

### 2.1 Survey Design

A cross‐sectional UK survey of clinicians with experience of managing SWHSI across a range of patient populations and surgical areas, described here according to the CHERRIES guidance for reporting results of internet surveys [8].

### 2.2 Survey Development

A survey development group composed of contributors with expertise in wound care, clinical trial design and management, and survey design was convened to determine the aims of the survey and outline the requirements for its design. Target group input and recommendations from previous engagement work were considered when designing the survey, ensuring that it met the needs and preferences of the intended population. This included considerations such as the number of questions to include, average likely completion time, and the nature of sociodemographic information to be collected.

The survey was conducted on the Qualtrics survey platform (Qualtrics, Provo, UT). Study information, including the voluntary nature of the study, was included at the start of the survey, followed by a consent to proceed. A contact details section was included at the end of the survey in case participants did want to be contacted about future research opportunities; however, this data was analysed separately from survey responses. As such, survey responses were anonymised at the point of analysis. All survey data was held securely within Hull York Medical School (University of Hull and University of York) systems, with password‐protected access restricted to study researchers.

In addition to sociodemographic information, three main areas of interest were identified for exploration: clinician perceptions of SWHSI, decision‐making in SWHSI and importance of clinical trial outcomes in SWHSI. The survey included five items related to respondent sociodemographic characteristics, 10 items relating to SWHSIs, and two items relating to contact information and future involvement over eight pages. All items included mandatory response validation except free text responses and contact information. A ‘back’ button was also included to allow respondents to go back to previous questions if necessary.

#### 2.2.1 Sociodemographic Characteristics

Participants were asked to give information regarding their professional role and specialty, geographical area of practice, healthcare setting and how commonly they are involved in the care of patients with SWHSI.

#### 2.2.2 Perceptions of SWHSI

Healing by secondary intention is utilised across many surgical areas. However, to understand the reasons driving decision‐making towards secondary intention healing, respondents were asked to indicate what they believed to be the most common reasons for choosing healing by secondary intention. Participants were asked about how commonly they encountered five main scenarios: dehiscence of a previously closed surgical wound, suspected or confirmed superficial infection of a surgical site, suspected or confirmed deep infection of a surgical site, inability to primarily close a surgical wound due to wound size and planned staged surgery in their practice. These scenarios were derived from previous work in the SWHSI population [6].

Response options were organised into a modified 4‐point Likert scale ranging from ‘common (more than 51% of the time)’ to ‘rare (less than 10% of the time)’, with an option for ‘I don't know’ also included for each scenario. The decision was made to include quantification of these terms as the options (‘common’, ‘somewhat common’, ‘uncommon’ and ‘rare’) were otherwise considered to be too subjective and open to variable interpretation.

Participants were also invited to submit any additional situations in which healing by secondary intention has been used and their perception of how common this situation is in their practice via a free text response question.

#### 2.2.3 Decision‐Making in SWHSI

Questions asking participants about their decision‐making around treatment of SWHSI, including the factors that guided them when deciding how to manage a SWHSI, were included in the second section.

Participants were asked to indicate the importance of eight individual factors in their decision‐making. These factors were: organisational difficulty in managing the SWHSI, potential deterioration in the patient's quality of life, costs of management to the patient, costs of management to the NHS, delivery of treatment in the outpatient setting, delays in discharge from inpatient care, rates of complications or reintervention and delays to other care. These options were derived from previous studies looking at SWHSI management and care [1, 9].

Participants could rate each factor on a five point modified Likert scale: ‘very important’, ‘somewhat important’, ‘neutral’, ‘somewhat unimportant’ and ‘very unimportant’. Participants were also invited to contribute any additional factors that affected their decision‐making that were not included in the survey in a free text section.

#### 2.2.4 Clinical Trial Outcomes in SWHSI

Respondents were asked to rate pre‐derived clinical trial outcomes according to their perceived importance in the context of wounds trials specifically. Commonly utilised clinical trial outcomes were identified to be included in the survey, organised according to outcome domains. These were based on the outcome domain taxonomy developed by Dodd et al. [10]. This ensured that a broad range of outcome types were considered by participants. Dodd et al. previously found that, of published core outcomes sets, while most (92%) included at least one physiological outcome, inclusion of quality of life or measure of functioning outcomes was less common (59%). Moreover, adverse events were less commonly included still (35%).

Five mortality and survival‐based outcomes were identified to be included in the survey: all‐ cause mortality at 7 days, all‐cause mortality at 30 days, death at ICU discharge, death at hospital discharge and all‐cause mortality at 1 year. Five physiological and clinical outcomes were included in the survey; wound healing at 30 days, pain score, surgical site infection presence, wound size and wound microbiology. Three life impact outcomes were included in the survey: general quality of life, wound‐specific quality of life and physical functioning in activities of daily living (ADLs). Three resource use outcomes were included in the survey: length of stay, cost of stay and need for further intervention. Three adverse event outcomes were included in the survey: death, required intervention to prevent permanent damage and serious medical events (e.g., severe allergic reactions, seizures, serious blood abnormalities), which would broadly comprise Clavien‐Dindo Grade III, IV and V complications [11]. Decisions on which outcomes to include in the survey were made by the development team with consideration given to their likely relevance to SWHSIs, however free‐text inputs were also included to ensure the broadest range of responses could be collected; if participants felt there were outcomes not listed in the survey that were very important or relevant, they were encouraged to submit those in the free‐text response areas.

The survey underwent two rounds of piloting amongst the wider Hull Academic Vascular Surgery Unit (AVSU) working group (consisting of three consultant vascular surgeons, six specialist nurses and six research fellows) to identify any operational issues, question ambiguity and evaluate the accessibility of the survey. Feedback received led to improvements in the clarity of participant information and survey questions (including optimisation of measurement scales used and inclusion of clear definitions of terms like ‘rare’, ‘uncommon’, etc.), inclusion of a wider range of professional roles and settings (e.g., expanding the range of nursing roles and clinical specialties included), and optimisation of free text items within the survey. Following completion of the pilot stage, the survey design was finalised for distribution (Appendix S1).

### 2.3 Survey Distribution

A recruitment and distribution strategy was devised by the survey development group, and in collaboration with the wider AVSU working group. Feedback from the target population was integrated to optimise the potential of reaching the widest sample of clinicians working with and treating patients with a SWHSI. Convenience sample methodology through professional channels was used to capture as broad a view of the area as possible.

Professional groups and organisations in surgery and wound care were targeted to deliver the survey via mailing lists to their memberships. This included distribution via the Vascular Society of Great Britain and Ireland (VSGBI), the Society of Vascular Nurses (SVN), the British Society for Dermatological Surgery (BSDS), the Association of Breast Surgery (ABS), WoundsUK, the Society of Tissue Viability (SoTV), the British Association of Plastic, Reconstructive and Aesthetic Surgeons (BAPRAS) and the Royal College of Surgeons of England through the Surgical Specialty Leads. The survey was also advertised on social media sites through the study accounts and website.

The survey was open from the 1st of September 2024 and closed on the 1st of November 2024. This was a voluntary survey; no incentives for completion were offered and participation in the survey was accepted as confirmation of informed consent. Reponses were collected anonymously and confidentially; participants were offered the option to leave contact information if they would like to be contacted about future research opportunities, however this information (where given) was separated from the dataset in order to maintain response anonymity. No randomisation or adaptive questioning was used as part of sampling potential survey participants.

### 2.4 Statistical Analysis

It was not possible to calculate view rates, participation rates or overall response rates due to the extensive nature of the survey visibility and the planned open distribution strategy. This was because we attempted to make the survey as openly available as possible. Any surveys with timestamps outside of the planned survey opening period were excluded.

The survey results are presented as counts and percentages of responses received for each question. Where surveys were returned incomplete in case of the participant closing the survey before the end, the data that were provided have been included in the results. All questions within the survey (except free text responses and contact information) were subject to mandatory response validation through the Qualtrics software; as such, it was not possible to partially answer any question. For this reason, there was no missing data requiring statistical correction through pairwise analysis or listwise deletion for any aspect of the survey—questions were either answered fully or not at all. Statistical analysis and data visualisation were completed using Qualtrics and Microsoft Excel.

### 2.5 Ethics Approval

The survey received a favourable opinion as part of the ethical review of the over‐arching SWHSI‐COS study (23/YH/0253; HRA and Health and Care Research Wales (HCRW)).

## 3 Results

During the survey period, 102 unique responses were received, with some attrition noted as the survey progressed. Respondents were drawn from a range of clinical professions and specialty practice areas (Table 1). The most common disciplines represented were surgeons (47%, 48/102), medical physicians (24%, 24/102) and advanced nursing practitioners (ANPs) (8%, 8/102). The most common specialty practice areas were vascular surgery (43%, 43/101), dermatological surgery (25%, 25/101) and tissue viability (15%, 15/101). Geographically, all regions of the United Kingdom were represented, with the majority of responses coming from clinicians working in Yorkshire (17%, 16/96), the South‐West of England (13%, 12/96) and the West Midlands (13%, 12/96). Most respondents practiced in specialist or tertiary care centres (54%, 61/114), followed by secondary care (34%, 39/114) and general practice (7%, 8/114); respondents to this section were able to select multiple settings to best represent their situations.

**TABLE 1:** Respondent information.

		Total responses	Survey completion
Professional background	Medical—Surgeon	48/102 (47.1%)	35/67 (52.2%)
	Medical—Physician	24/102 (23.5%)	15/67 (22.4%)
	Nursing—Advanced practitioner	8/102 (7.8%)	6/67 (9%)
	Nursing—Research	6/102 (5.9%)	4/67 (6%)
	Nursing—Community/District	4/102 (3.9%)	2/67 (3%)
	Nursing—Ward‐based	1/102 (1%)	0
	Nursing—Outpatient	1/102 (1%)	0
	Industry	3/102 (2.9%)	1/67 (1.5%)
	Researcher—non‐clinical	1/102 (1%)	0
	Other	6/102 (5.9%)	4/67 (6%)
Clinical specialty	Vascular surgery	43/101 (42.6%)	32/67 (47.8%)
	Dermatology/dermatological surgery	25/101 (24.8%)	17/67 (25.4%)
	Tissue viability	15/101 (14.9%)	10/67 (14.9%)
	Breast surgery	3/101 (3%)	1/67 (1.5%)
	Infectious diseases	2/101 (2%)	1/67 (1.5%)
	Cardiothoracic surgery	1/101 (1%)	1/67 (1.5%)
	Colorectal surgery	1/101 (1%)	1/67 (1.5%)
	Trauma and orthopaedic surgery	1/101 (1%)	0
	Other	10/101 (9.9%)	5/67 (7.5%)
Geographical location	England—Yorkshire	16/96 (16.7%)	12/67 (17.9%)
	England—West Midlands	12/96 (12.5%)	5/67 (7.5%)
	England—South West	12/96 (12.5%)	8/67 (11.9%)
	England—South East	9/96 (9.4%)	6/67 (9%)
	England—East of England	9/96 (9.4%)	7/67 (10.4%)
	England—North West	9/96 (9.4%)	7/67 (10.4%)
	Scotland	8/96 (8.3%)	7/67 (10.4%)
	England—East Midlands	8/96 (8.3%)	5/67 (7.5%)
	England—North East	7/96 (7.3%)	6/67 (9%)
	England—London	3/96 (3.1%)	1/67 (1.5%)
	Wales	2/96 (2.1%)	2/67 (3%)
	Northern Ireland	1/96 (1%)	1/67 (1.5%)
Practice setting (respondents may select multiple responses)	Specialist hospital/tertiary care	61/114 (53.5%)	44/91 (48.4%)
	District general hospital/secondary care	39/114 (34.2%)	27/91 (29.7%)
	General practice/primary care	8/114 (7%)	6/91 (6.5%)
	Research—university/lab‐based	7/114 (6.1%)	5/91 (5.5%)
	Private practice	7/114 (6.1%)	4/91 (4.4%)

	Other community setting	5/114 (4.4%)	4/91 (4.4%)
Industry/commercial	2/114 (1.8%)	1/91 (1.1%)
Frequency of encountering SWHSIs	Most of the time (more than once per day)	18/76 (23.7%)	17/67 (25.4%)
	Often (more than once per week)	33/76 (43.4%)	28/67 (41.8%)
	Sometimes (more than once per month)	14/76 (18.4%)	12/67 (17.9%)
	Rarely (less than once per month)	8/76 (10.5%)	8/67 (11.9%)
	Never	3/76 (3.9%)	2/67 (3%)

Most respondents reported seeing patients with SWHSI ‘often (more than once per week)’ (43%, 33/76) or ‘most of the time (more than once per day)’ (24%, 18/76).

### 3.1 Perceptions of SWHSI

Suspected or confirmed superficial infection as a cause of SWHSI showed a marked split in responses, with 50% of respondents considering this to be a rare (35.5%, 27/76) or uncommon (14.5%, 11/76) reason, and 46% of respondents considering it to be somewhat common (31.6%, 24/76) or common (14.5%, 11/76) (Figure 1). When analysed according to professional group, the largest group of respondents, surgeons, were split evenly between these viewpoints (50% (19/38) selected common (18.4%, 7/38) or somewhat common (31.6%, 12/38), and 50% (19/38) selected uncommon (21.1%, 8/38) or rare (28.9%, 11/38)); this primarily reflected the views of vascular surgeons, who made up 82% (31/38) of respondents in the surgical group. The second largest group, physicians (91.3% of whom reported working in dermatology or dermatological surgery) overwhelmingly selected the rare option (89.5%, 17/19). However, when accounting for respondents' familiarity with SWHSIs across all professional groups, 70.6% of respondents who reported encountering a SWHSI daily or weekly considered suspected or confirmed superficial infection to be a rare (43.1%, 22/51) or uncommon (27.5%, 14/51) cause of SWHSI.

**FIGURE 1:**
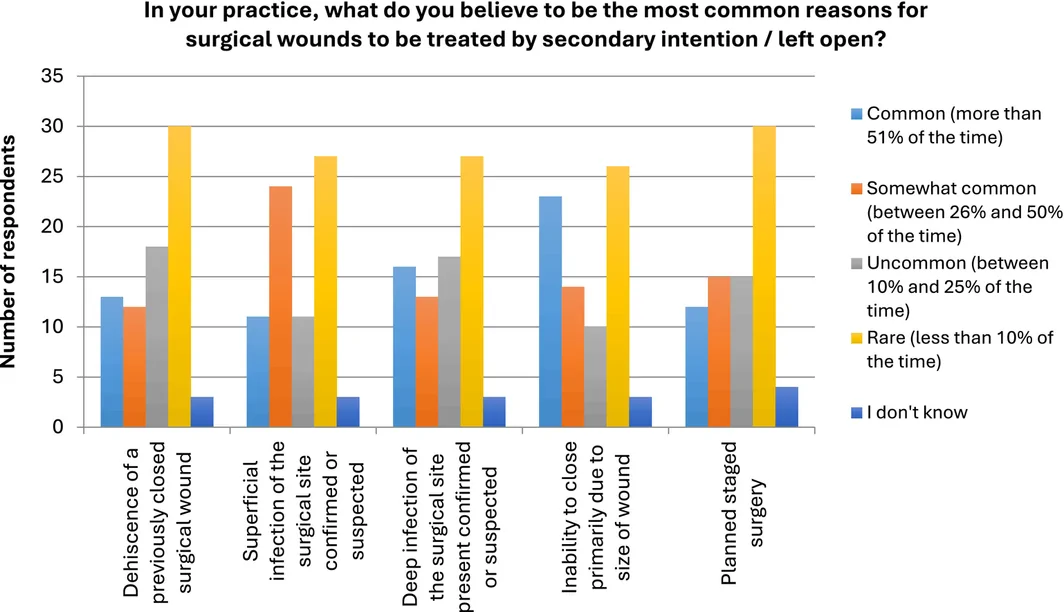
Perceptions of SWHSI—reasons for management by secondary intention amongst survey respondents.

Similarly, inability to close a wound primarily due to wound size showed a marked split amongst respondents, with around half of respondents overall (47.6%, 36/76) selecting rare (34.2%, 26/76) or uncommon (13.2%, 10/76), and around half (48.7%, 37/76) selecting common (30.3%, 23/76) or somewhat common (18.4%, 14/76). Familiarity with SWHSIs did not have an impact on this perception, with just over half (52.9%, 27/51) of respondents who reported encountering a SWHSI daily or weekly selecting the rare (35.3%, 18/51) or uncommon (17.6%, 9/51) option. However, when perceptions were analysed according to professional group, a majority of surgeons selected rare or uncommon (60%, 23/38), while this trend was reversed amongst physicians, with 59% (10/17) selecting the common or somewhat common options. Amongst respondents in nursing roles, 53% (10/19) selected the common or somewhat common option.

Suspected or confirmed deep infection was considered a rare (35.5%, 27/76) or uncommon (22.4%, 17/76) reason for SWHSI by most respondents across professional groups. Similarly, planned healing by secondary intention or staged surgery was considered rare (39.5%, 30/76) or uncommon (19.7%, 15/76) by over half of respondents (59.2%, 45/76) across professional groups. However, participants who reported seeing SWHSIs daily or weekly did show a slight preference for the uncommon (21.6%, 11/51) or rare (35.3%, 18/51) options.

Finally, dehiscence of a previously primarily closed surgical wound was considered a rare cause of SWHSI, with most participants across professional groups and across familiarity levels with SWHSI selecting ‘rare’ (39.5%, 30/76) or ‘uncommon’ (24%, 18/76).

Respondents were also asked to give any other reasons they had seen in their practice for choosing to manage a surgical wound by secondary intention. Fourteen free‐text responses were received, and highlighted that in some cases, a better cosmetic result was expected from healing by secondary intention (29%, 4/14). Others reiterated concerns regarding deep or superficial infection risk, and the need to identify devitalised or ischaemic tissue promptly in the postoperative period to reduce this risk (50%, 7/14); specific examples of lower limb fasciotomies, deep anastomotic dehiscence and diabetic foot sepsis were given. Difficulty in achieving tension‐free closure was suggested by two respondents (14%). Finally, a concern regarding the availability of community nursing care was also noted (7%).

### 3.2 Decision‐Making for SWHSI

Most respondents reported that the likelihood of complications or reinterventions (89.5%, 68/76), potential deterioration in patient quality of life (84.2%, 64/76), and how treatment would be delivered in the outpatient setting (86.9%, 66/76) were either very important or somewhat important considerations when they were deciding whether to manage a wound by secondary intention (Figure 2). Delays to other care or delays to discharge from inpatient care also scored highly with 67.1% (51/76) and 64.5% (49/76), respectively, indicating that these factors were either very important or somewhat important.

**FIGURE 2:**
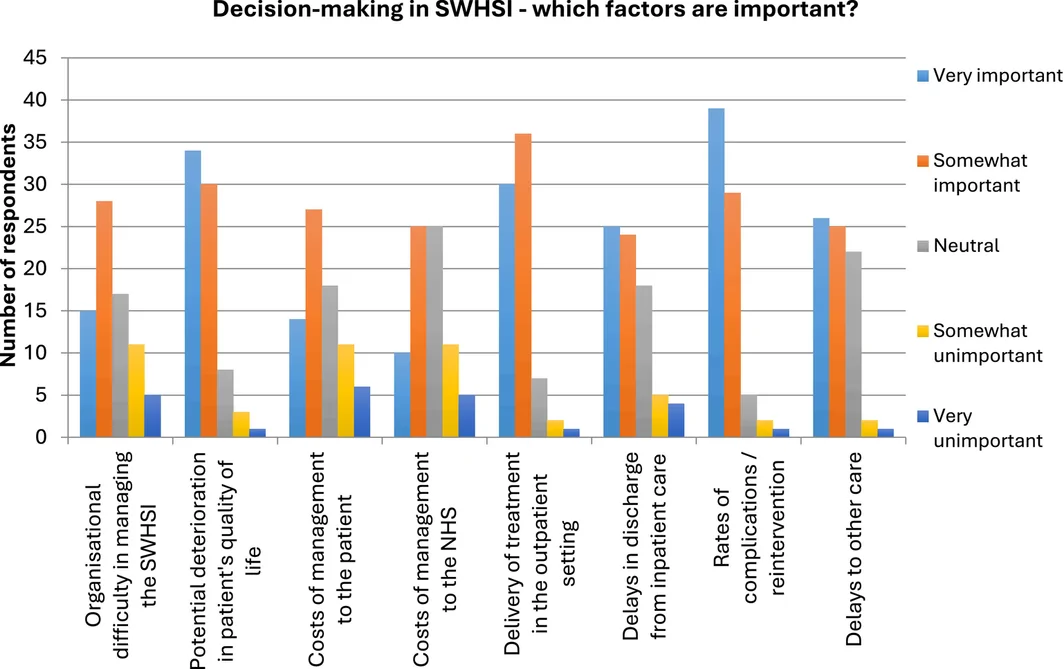
Decision‐making in SWHSI—importance of associated factors in treatment choice.

Organisational difficulty in managing a SWHSI, cost of SWHSI management to the patient and cost of SWHSI management to the health service scored less highly in the top two categories, with 56.6% (43/76), 53.9% (41/76) and 46.1% (35/76) marking these as very important or somewhat important to their decision‐making. These findings showed no notable variation amongst professional groups; however, when looking particularly at responses from participants who reported seeing SWHSIs daily or weekly in their practice, delivery of care in the outpatient setting was most commonly placed in the very important or somewhat important category (90.2%, 46/51), followed by potential deterioration in patient quality of life (82.4%, 42/51), and delays to discharge from inpatient care (64.7%, 33/51).

Eight free‐text responses were received regarding any other factors that may impact decision‐making; factors offered here included patient preference, risk of delay in fitting prostheses in amputee patients, risks of arterial graft infection or fistulation and difficulty in tolerating and doing dressing changes. Multiple participants noted the importance of a holistic, multidisciplinary approach to decision‐making.

Social and service‐level factors reported as relevant included consideration of funding availability for dressings and additional services needed, the level of impact on working‐age patients specifically, and local team preferences with a particular emphasis on differences between surgical specialty teams and tissue viability services. Consideration of how patients might cope with adjunctive therapies like negative pressure wound therapy dressings (and the possible additional risks of those, including trip hazards) was also mentioned.

### 3.3 Clinical Trial Outcomes in SWHSI

Participants were asked their opinions about the importance of a range of outcomes commonly utilised in clinical trials in wound care (summarised in Table 2).

**TABLE 2:** Importance of outcomes—responses.

Outcome	Response count (percentage)				
	Very important	Somewhat important	Neutral	Somewhat unimportant	Very unimportant
Mortality and survival‐based outcomes (67 total responses)					
All‐cause mortality—7 days	24 (35.8%)	10 (14.9%)	14 (20.9%)	8 (11.9%)	11 (16.4%)
All‐cause mortality—30 days	29 (43.3%)	20 (29.9%)	9 (13.4%)	3 (4.5%)	6 (9%)
All‐cause mortality—1 year	27 (40.3%)	20 (29.9%)	9 (13.4%)	5 (7.5%)	6 (9%)
Death at hospital discharge	24 (35.8%)	17 (25.4%)	12 (17.9%)	5 (7.5%)	9 (13.4%)
Death at ICU discharge	20 (29.9%)	10 (14.9%)	17 (25.4%)	8 (11.9%)	12 (17.9%)
Physiological and clinical outcomes (67 total responses)					
Wound microbiology	17 (25.4%)	38 (56.7%)	11 (16.4%)	0	1 (1.5%)
Wound size	31 (46.3%)	32 (47.8%)	4 (6.4%)	0	0
Pain score	39 (58.2%)	24 (35.8%)	4 (6%)	0	0
Wound healing at 30 days	42 (62.7%)	25 (37.3%)	0	0	0
Surgical site infection presence	47 (70.1%)	19 (28.4%)	1 (1.5%)	0	0
Life impact outcomes (67 total responses)					
Physical functioning in activities of daily living (ADLs)	48 (71.6%)	17 (25.4%)	2 (3%)	0	0
General quality of life	51 (76.1%)	15 (22.4%)	0	1 (1.5%)	0
Wound‐specific quality of life	53 (79.1%)	13 (19.4%)	1 (1.5%)	0	0
Resource‐use outcomes (67 total responses)					
Cost of stay	28 (41.8%)	25 (37.3%)	7 (10.4%)	5 (7.5%)	2 (3%)
Length of stay	34 (50.7%)	23 (34.3%)	7 (10.4%)	1 (1.5%)	2 (3%)
Need for further intervention	39 (58.2%)	21 (31.3%)	6 (9%)	0	1 (1.5%)
Adverse‐events outcomes (67 total responses)					
Serious medical events—e.g., severe allergic reactions, seizures, serious blood abnormalities	37 (55.2%)	19 (28.4%)	6 (9%)	3 (4.5%)	2 (3%)
Required intervention to prevent permanent damage	42 (62.7%)	15 (22.4%)	5 (7.5%)	2 (3%)	3 (4.5%)
Death	48 (71.6%)	8 (11.9%)	5 (7.5%)	2 (3%)	4 (6%)

#### 3.3.1 Mortality and Survival‐Based Outcomes

All‐cause mortality was rated highly amongst respondents with 73% (49/67) selecting this outcome as very important or somewhat important at 30 days and 70% (47/67) at 1 year. Of the other outcomes, death at hospital discharge was selected as very important or somewhat important by 61% (41/67), all‐cause mortality at 7 days by 51% (34/67) and death at ICU discharge by 45% (30/67). There were no prominent trends noted when accounting for professional background or how often respondents reported seeing SWHSI patients in their usual practice.

#### 3.3.2 Physiological and Clinical Outcomes

All respondents selected wound healing at 30 days to be either very important (63%, 42/67) or somewhat important (37%, 25/67); presence of surgical site infection also scored very highly, with 70% of respondents selecting very important (47/67), and a further 28% (19/67) selecting somewhat important for this outcome. 94% of respondents rated pain score (63/67) and wound size (63/67) as very important or somewhat important. Finally, 82% of respondents selected wound microbiology as very important or somewhat important (55/67). Again, there were no prominent trends noted when accounting for professional background or how often respondents reported seeing SWHSI patients in their usual practice.

#### 3.3.3 Life Impact Outcomes

All life impact outcomes were rated highly for importance. 99% rated general quality of life (66/67) and wound‐specific quality of life (66/67) as very important or somewhat important; 97% (65/67) rated physical functioning in ADLs similarly.

#### 3.3.4 Resource‐Use Outcomes

Need for further intervention was rated as very important or somewhat important by 90% of respondents (60/67), length of stay by 85% (57/67) and cost of stay by 79% (53/67). There were no trends noted when accounting for professional background or how often respondents reported seeing SWHSI patients in their usual practice.

#### 3.3.5 Adverse‐Events Outcomes

85% of respondents (57/67) rated a need for reintervention to prevent permanent damage as either very or somewhat important. The other listed outcomes also scored highly, with 84% (56/67) rating death and serious medical event (e.g., severe allergic reaction, seizures, serious blood abnormalities) as very or somewhat important. There were no prominent trends noted when accounting for professional background or how often respondents reported seeing SWHSI patients in their usual practice.

#### 3.3.6 Other Outcomes

Respondents were asked to give any additional outcomes that they felt were important in SWSHI assessment and management, that were not otherwise included in the survey. Eleven free‐text responses were received. Additional outcomes suggested included cost‐effectiveness, cosmetic satisfaction after healing, and therapy adherence or concordance. Additionally, the presence of antimicrobial resistance and diversity of microbial presence in the wound—outcomes not routinely measured by NHS microbiology laboratories—were suggested.

## 4 Discussion

Regarding perceptions of SWHSIs, concerns about the ability to close the wound primarily and the presence or suspicion of superficial wound infection were considered the most common reasons for managing a surgical wound by secondary intention.

Though opinion was somewhat split amongst respondents, this is consistent with the current understanding of SWHSI as a broad clinical entity, with notable differences in primary pathology (i.e., the pathology treated by the surgery leading to the SWHSI) leading to diverse perceptions of its necessity, benefits and disadvantages. A qualitative study by McCaughan et al. explored clinician's experiences of SWHSI, and reported the main types of open surgical wounds encountered in the participant's clinical practice [6]. These included extensive abdominal cavity wounds following laparostomy or wound dehiscence and large open wounds following diabetic foot debridement, both of which may often result in insufficient local healthy, viable tissue for primary closure. Other common SWHSI types reported in the McCaughan study included pilonidal sinus and dehisced lymphadenectomy wounds in the groin or axilla. These wounds are likely to be more limited in size, leading to less concern about the technical possibility of primary closure (particularly given the various flap techniques commonly used to treat pilonidal sinuses [12]), but a higher concern regarding surgical site infection, given their anatomical location and high risk of contamination [13].

Additionally, a large proportion of respondents in our survey reported working primarily in dermatology or dermatological surgery, with Mohs micrographic procedures for treatment of skin cancers a common cause of SWHSI in this area [14]. Studies in this area suggest that cosmesis was likely to be the primary concern when choosing secondary intention healing postoperatively [15]. This is consistent with the free‐text responses received in our survey, with multiple participants noting that in some circumstances within their patient population, healing by secondary intention was considered to lead to better cosmetic outcomes than primary closure.

In exploring the factors that affected respondents' choices when deciding how to manage a surgical wound, concerns about the likelihood of complications, particularly those requiring reintervention, were salient in the survey population. Concerns regarding a higher risk of wound complication requiring readmission to hospital and possible reintervention in SWHSIs are considerable; a 2019 cohort study of patients with SWHSIs reported a hospital readmission rate of 24% and return to theatre rate of 16.8%, compared to a 30‐day emergency readmission rate of 6.6% across all NHS patients [2, 16]. More recently, the SWHSI‐2 multicentre randomised controlled trial, which compared outcomes from negative pressure wound therapy (NPWT) or usual care in 686 patients with SWHSI, reported high complication rates across both treatment arms; hospital admission rates were reported at 19.7% and 18.1% in the intervention and control arms respectively [17]. Reoperation rates (24.4% and 21.6% respectively), and wound infection rates (31.9% and 31.2%) were also high across treatment arms.

However, robust studies comparing outcomes following primary closure to secondary intention healing are scarce; they are mostly limited to observational designs, and the results reported are mixed. Higher reported rates of complications in studies like SWHSI‐2, as well as the general perception of SWHSIs as being more vulnerable to complications, may be reflective of the increased underlying complexity of the wounds themselves, rather than increased risk from management by secondary intention healing—in effect, clinicians may be more likely to choose secondary intention healing in cases where primary closure is challenging or not possible, or where there is already suspected or proven infection present; this consideration is somewhat supported by our survey findings, with high proportions of respondents reporting these specific incidences to be common reasons for management by secondary healing.

Amongst patients undergoing emergency laparotomy, there was no significant difference found in surgical site infection rates between open and closed skin management strategies in one cohort study, though participants were not randomised, resulting in significant heterogeneity between participant groups [18]. Similarly, one randomised study comprising 49 participants found no difference in wound complication rates between participants receiving negative pressure wound therapy in open or closed contaminated and dirty surgical wounds [19]. However, in contrast, a cohort study of patients undergoing Mohs micrographic surgery or wide local excision for treatment of skin cancer found a significantly higher infection rate associated with secondary intention healing [20]. Ultimately, as high‐quality comparative randomised studies accounting for the large and complex variation in risk between different procedure types and patient cohorts are not available, it is not possible to conclusively evaluate the validity of the perception that secondary intention healing may have a higher risk of wound complications leading to readmission or reintervention; however, our survey suggests that this clinically‐grounded perception is pervasive and does impact decision‐making around surgical‐planning and wound management.

Impact on quality of life was also highlighted as an important consideration when considering management by secondary intention, as well as the logistical requirements of managing a SWHSI in the outpatient setting. This concern is consistent with patient‐reported experiences of living with SWHSIs, with previous qualitative studies reporting wide‐ranging impacts of SWHSI on quality of life, including on mental and physical health, as well as more wide‐ranging impacts on mobility, sleep quality, social functioning, financial wellbeing and general decline associated with a long recovery process [4, 21]. Participants in these studies also highlighted the impact of outpatient management on their quality of life, particularly the high burden of interaction with healthcare services, often requiring multiple daily or weekly interactions for assessment, dressing changes and other care needs [3]. Quality of life evaluation is also necessary for cost‐effectiveness analysis.

Delays to other care or delays to discharge were considered important factors, as were costs associated with managing a SWHSI to the patient and the health service to a lesser degree. The costs associated with SWHSI can be consequential. Guest et al., reported the cost of an unhealed surgical wound (i.e., dehisced primarily closed wound or wound for planned secondary intention healing) was approximately £7345 per wound over 12 months of outpatient management, with the overall mean cost of an unhealed wound at 12 months likely double that of a healed wound (£13 682 vs. £5997) [9]. Additionally, the longer a wound takes to heal, if it heals at all, the higher the cost of management—the monthly cost to the NHS of managing the wound begins to climb from around month 6 in a non‐healing wound [9]. Additionally, some patients with lived experience of SWHSIs have reported personal financial impacts, stemming from increased costs of transportation due to inability to drive to appointments, or from reduced income due to inability to work [4].

Of the commonly utilised trial outcomes, respondents considered all‐cause mortality at 30 days and 1 year to be the most important mortality outcome measures for the SWHSI population. Of the clinical and physiological outcomes, wound healing at 30 days and presence of surgical site infection were considered the most important with close to unanimous agreement. Pain score and wound size also rated highly. Of life impact outcomes, generic and wound‐specific quality of life were considered the most important, followed by physical functioning in activities of daily living. Length of hospital stay and need for reintervention were both rated highly of the resource use outcomes; cost of stay was also rated highly but this did contradict our earlier findings where only 46% of participants considered the cost of care to the NHS to be an important factor in their decision‐making around SWHSIs. Additionally, as a large proportion of care, and therefore care costs, for SWHSI are likely to be in the outpatient setting, it is unlikely that the cost of hospital stay alone will account for the costs incurred in SWHSI treatment; as such, a more comprehensive and practical accounting of resource use may be necessary. Finally, of the adverse outcomes listed, all three outcomes were rated highly with a requirement for reintervention to prevent permanent damage considered the most important overall. These findings are broadly consistent with qualitative studies in this area, though these are scarce [6, 22].

While these findings provide a valuable insight into the priorities of the clinical team in managing SWHSIs, there is little data available regarding how these priorities compare with those of the patient population who experience these wounds. Further direct exploration of patient priorities is needed to understand the views of this stakeholder group and explore any contradiction or concordance in priorities between the clinicians providing care and the patients receiving it.

### 4.1 Strengths and Limitations

This survey provided valuable and previously unexplored insights into the perceptions and decision‐making processes of clinicians working with SWHSI. There are, however, some limitations to the scope of the survey that may impact the generalisability of the results.

As the survey was openly advertised via various newsletters, websites, mailing lists and word of mouth, it is difficult to calculate the number of potential participants that may have been reached who declined to participate and the resulting response rate. Thus, it is not possible to estimate the potential participants for response bias. Beyond this, clinicians with a medical background were strongly represented over other groups, and primarily from the disciplines of vascular surgery, and dermatology and dermatological surgery. This may reflect the salience of the topic to clinicians working in this area, as these specialties commonly utilise secondary intention healing postoperatively in comparison to other specialties. However, other clinical specialties who commonly manage SWHSIs were severely underrepresented in the participant population—in particular, colorectal surgery and plastic surgery who, together with vascular surgery, make up the majority of operations resulting in SWHSI [2], and the views of tissue viability, district and community nursing colleagues, who provide the majority of direct wound care and monitoring postoperatively. This may limit the generalisability of our findings to the cross‐specialty SWHSI cohort.

Additionally, while the survey included a varied range of commonly utilised trial outcomes, this selection was certainly not exhaustive, and in some aspects may have been too narrow in its scope, for example when considering the outcome ‘wound healing at 30 days’ rather than ‘wound healing’ generally. The inclusion of free text spaces was essential in allowing a broad range of opinions to come through as participants were free to add their own outcomes, and include comments or clarifications.

### 4.2 Directions for Future Research

This study forms an essential component of the wider SWHSI‐COS Study, which aims to develop a core outcome set for SWHSI. In the next phase, these survey findings will be combined with further insights from direct patient experience and a systematic review of clinical trials in SWHSI, and undergo a modified Delphi process to identify a core set of outcomes to be reported in future SWHSI trials. This standardisation will help to ensure more consistent and reliable outcome reporting and significantly impact future research in this clinical area.

## 5 Conclusion

This study provides a direct insight into the priorities of UK clinicians in managing SWHSIs, including current clinical perceptions of SWHSI, how clinicians are making decisions about managing SWHSIs, and the views of the surveyed clinical community regarding which outcomes are most important to measure in future clinical trials in this area.

## Funding

M.S. is funded by the National Institute of Health and Social Care (NIHR) Doctoral Fellowship NIHR303688. The views expressed are those of the author(s) and not necessarily those of the NIHR or the Department of Health and Social Care. Early development of this study was funded by a Clinical Research Fellowship awarded to M.S. by Hull University Teaching Hospitals NHS Trust.

## Ethics Statement

This study received ethical approval from the NHS Health Research Authority (HRA) as a part of the over‐arching SWHSI‐COS Study (IRAS: 327080). It was reviewed by Yorkshire and the Humber—Leeds West Research Ethics Committee (23/YH/0253). The study was sponsored by Hull University Teaching Hospitals NHS Trust.

## Conflicts of Interest

The authors declare no conflicts of interest.

## Supporting information


**Appendix S1:** SWHSI‐COS Clinician Survey

## Data Availability

The data that support the findings of this study are available from the corresponding author upon reasonable request.
