# Supplementary material for: National Clinician Survey of Outcomes for Trials in Surgical Wounds Healing by Secondary Intention (SWHSI)
Source: Int Wound J. 2026 Oct 20;23(10):e71066. doi: 10.1111/iwj.71066 (PMC13682952; doi:10.1111/iwj.71066)
Supplement: Supplementary file 1 [file IWJ-23-e71066-s001.pdf]

Thank you for your interest in the SWHSI-COS project!

This survey takes around 10-15 minutes to complete. We recommend completing it on a computer.

This survey is about designing clinical trials in wounds research.

In particular, we are looking at surgical wounds healing by secondary intention (also called open surgical wounds or SWHSIs)

To design trials well, we need to make sure that they measure the right things. This survey explores your opinions on which outcome measures are most important in SWHSI trials. This survey is open to **UK-based researchers and clinicians** who work with patients with open surgical wounds or SWHSIs.

All answers are recorded anonymously, and we don't collect any personal data about you. If you would like to be contacted about this study in the future, there is a space to submit your contact details at the end of the survey, however this information will be stored separately from your survey answers.

The SWHSI-COS project is one part of the SWHSI-3 Study. More information can be found at our website.

The SWHSI-3 Study is managed from the Academic Vascular Surgery Department at Hull York Medical School. This study is funded by the NIHR Doctoral Fellowship Programme (NIHR303688). The views expressed are those of the author(s) and not necessarily those of the NIHR or the Department of Health and Social Care.

Consent

By completing this questionnaire I agree that:

- I am voluntarily offering my views, I understand that I do not have to take part, and I can stop at any time
- My comments may be anonymously quoted in reports or publications reporting on the results of this survey and associated work
- The research team will have access to my contact information only if I provide this at the end of the survey (note: contact information is accessed separately from survey responses)

○ Continue to survey

○ I do not wish to continue

Professional experience with SWHSIs

So we can understand your professional experience and background working with patients who have SWHSIs, please provide the following information.

Professional role: what best describes your professional role?

What is your professional role?

Clinical specialty: what best describes your main clinical area of practice?

What is your main clinical area of practice?

Location: where do you mainly practice?

[▾]

Which healthcare setting do you work in? - please select all that apply

- ☐ General Practice / Primary care
- ☐ District General Hospital / Secondary care
- ☐ Specialist Hospital / Tertiary care
- ☐ Other Community Setting
- ☐ Industry - Commercial
- ☐ Research - University / Lab-based
- ☐ Private Practice
- ☐ [ ] Other, please specify:

How often do you see patients with SWHSIs in your professional practice?

- ○ Never
- ○ Rarely - less than 1 per month
- ○ Sometimes - more than 1 per month
- ○ Often - more than 1 per week
- ○ Most of the time - more than 1 per day

In your practice, what do you believe to be the most common reasons for surgical wounds to be treated by secondary intention / left open?

	Common (more than 51% of the time)	Somewhat common (between 26% and 50% of the time)	Uncommon (between 10% and 25% of the time)	Rare (less than 10% of the time)	I don't know
Dehiscence of a previously closed surgical wound	○	○	○	○	○
Superficial infection of the surgical site confirmed or suspected	○	○	○	○	○
Deep infection of the surgical site present confirmed or suspected	○	○	○	○	○
Inability to close primarily due to size of wound	○	○	○	○	○
Planned staged surgery	○	○	○	○	○

Have you seen any other reasons for leaving surgical wounds to heal by secondary intention in your practice? Please briefly tell us what they are and how common you believe them to be:

Please tell us how important the following concerns and considerations are to you when deciding how to manage a SWHSI in your clinical practice

	Very important	Somewhat important	Neutral	Somewhat unimportant	Very unimportant
Organisational difficulty in managing the SWHSI	○	○	○	○	○
Potential deterioration in patient's quality of life	○	○	○	○	○
Costs of management to the patient	○	○	○	○	○
Costs of management to the NHS	○	○	○	○	○
Delivery of treatment in the outpatient setting	○	○	○	○	○
Delays in discharge from inpatient care	○	○	○	○	○
Rates of complications / reintervention	○	○	○	○	○
Delays to other care	○	○	○	○	○

Do you have any additional concerns about SWHSIs that haven't been included in this survey? Please briefly tell us what they are, and how important they are to your decision making:

In this section, we'd like to explore your opinions about the existing outcome measures used in wound research trials.

In clinical trials, these are usually listed as the primary or secondary outcomes of the trial and should be carefully chosen to make sure the results of the trial are relevant, useful and usable in clinical decision-making and service design.

Please tell us how important you believe the outcome is to include in a clinical trial.

At the end we ask for any other outcomes that you believe are important to measure in this population that we haven't mentioned previously.

Mortality and survival based outcomes

	Very important	Somewhat important	Neutral	Somewhat unimportant	Very unimportant
All cause mortality at 7 days	○	○	○	○	○
All cause mortality at 30 days	○	○	○	○	○
Death at ICU discharge	○	○	○	○	○
Death at hospital discharge	○	○	○	○	○

	Very important	Somewhat important	Neutral	Somewhat unimportant	Very unimportant
All cause mortality at 1 year					

Physiological and clinical outcomes

	Very important	Somewhat important	Neutral	Somewhat important	Very unimportant
Wound healing at 30 days					
Pain score					
Surgical site infection presence					
Wound size					
Wound microbiology					

Life impact outcomes

	Very important	Somewhat important	Neutral	Somewhat unimportant	Very unimportant
General quality of life					
Wound-specific quality of life					
Physical functioning in activities of daily living (ADLs)					

Resource-use outcomes

	Very important	Somewhat important	Neutral	Somewhat unimportant	Very unimportant
Length of stay	○	○	○	○	○
Cost of stay	○	○	○	○	○
Need for further intervention	○	○	○	○	○

Adverse events outcomes

	Very important	Somewhat important	Neutral	Somewhat unimportant	Very unimportant
Death	○	○	○	○	○
Required intervention to prevent permanent damage	○	○	○	○	○
Serious medical events - e.g. severe allergic reactions, seizures, serious blood abnormalities	○	○	○	○	○

Are there any other outcomes you think are important to measure in the population of patients with SWHSIs? Please tell us briefly what they are, and how important you believe them to be:

Thank you for taking part in this survey.

If you would be interested in the next stage of the SWHSI-COS project, please leave your contact details below. Please note, this section will remain separate from your survey answers.

Otherwise, please click submit.

Name

Job Title

Organisation

Email

Twitter Handle (optional)

Telephone (optional)

Would you be interested in being part of a future Delphi study about core outcome set development for SWHSI?

- ○ Yes, please contact me
- ○ I'm not sure, but I'd like to know more about it
- ○ No, thank you

Powered by Qualtrics
